# Efficient Automatic Pollen Recognition From Fossil Pollen Samples: A High‐Resolution Example Record From Palaeolake Kieshofer Moor, Northeastern Germany

**DOI:** 10.1002/ece3.73866

**Published:** 2026-06-21

**Authors:** Martin Theuerkauf, Alexander Gillert

**Affiliations:** ^1^ Institute of Ecology Leuphana University Lüneburg Lüneburg Germany; ^2^ Greifswald Mire Centre, Institute of Botany and Landscape Ecology University of Greifswald Greifswald Germany; ^3^ Ecology, Behavior and Evolution Department University of California San Diego La Jolla California USA

**Keywords:** convolutional neural networks, Holocene, Lateglacial, pollen analysis, TOFSI, vegetation history

## Abstract

Pollen analysis is a crucial tool for reconstructing past vegetation and ecosystems. Until now, pollen analysis has been a time‐consuming manual process, severely limiting the number of records an analyst can produce and their temporal resolution. Recently, automatic approaches based on artificial neural networks have shown potential for classifying multiple pollen types. These approaches performed well with clean, modern reference material, but not with real‐world fossil pollen samples from, for example lake sediments. To overcome this limitation, our TOFSI approach uses two neural networks to first detect and then classify pollen and other objects. Here, we apply the approach, for the first time, to a long lake sediment sequence at a very high resolution of 1 cm. To this end, a model has been trained to recognise 48 pollen, spore and NPP classes. Our approach performs excellently for the classes that are well represented in the training data. At the 0.5 confidence level, the automatic recognition achieves a recall and precision of at least 0.9. However, performance tends to decline for classes with fewer than ~100 training objects. We conclude that, when suitable images and model training are provided, TOFSI can accurately detect and classify multiple pollen, spores and NPP classes in lake sediment samples. The approach hence allows fully automated analysis when limited taxonomic resolution is sufficient. When full taxonomic resolution is required, TOFSI can be used in a semi‐automatic approach involving manual revision of critical objects. Both approaches substantially reduce analysis times, while the resulting count sums and, consequently, the statistical reliability of the results are often much higher. Besides improved productivity, an image‐based workflow could offer palynologists several practical improvements, including simplified student training and communication between researchers. Extended documentation and long‐term storage of results may improve the standardisation of pollen counts.

## Graphical abstract

We applied the two‐stage, AI‐based TOFSI approach to test automatic pollen recognition with fossil pollen samples. The algorithm performs very well for all major pollen types and other non‐pollen object classes, suggesting that such tools have the potential to substantially increase the efficiency of pollen analysis.

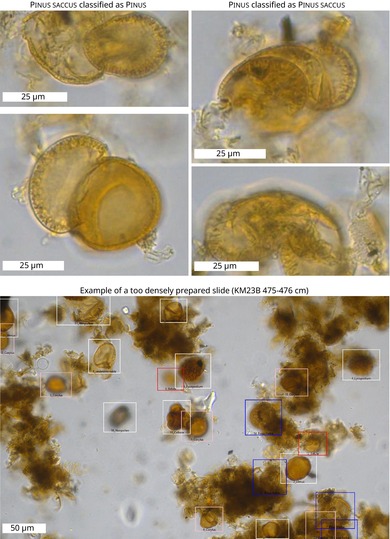

## ZUSAMMENFASSUNG

Die Pollenanalyse ist ein wichtiges Werkzeug zur Rekonstruktion von Vegetationen und Ökosystemen in der Vergangenheit. Pollenanalysen werden bisher ausschließlich manuell durchgeführt. Sie sind sehr zeitaufwändig: Die Anzahl der Proben, die eine Person bearbeiten kann, ist daher sehr beschränkt. Jüngste Arbeiten zeigen, dass automatische Verfahren auf Basis künstlicher neuronaler Netze durchaus zahlreiche Pollentypen unterscheiden können. Allerdings liefern diese Verfahren bisher gute Ergebnisse nur mit sauberem, modernem Referenzmaterial, nicht mit realen fossilen Pollenproben, beispielsweise aus Seesedimenten. Für eine bessere Erkennung kombiniert unser TOFSI‐Ansatz zwei Netzwerke – das erste detektiert zunächst Pollen, Sporen und andere Objekte, das zweite klassifiziert dann die gefundenen Objekte. Hier wenden wir den Ansatz erstmals an Proben aus Seesedimenten, mit einer sehr hohen Auflösung von 1 cm, an. Dazu wurde ein Modell für die Erkennung von 48 Pollen‐, Sporen‐ und NPP‐Klassen trainiert. Die manuelle Überprüfung der Ergebnisse und der Vergleich mit einem manuell gezählten Pollendiagramm vom gleichen Standort zeigen eine sehr gute Erkennung für alle Klassen, die in den Trainingsdaten zahlreich vertreten sind. Bei einem Konfidenzniveau von 0.5 liegen precision und recall über 0.9. Bei Klassen mit weniger als ~100 Trainingsobjekten ist die Erkennung schlechter. Mit geeigneten Bildern und einem passend trainierten Modell kann TOFSI zahlreiche Pollen‐, Sporen‐ und NPP‐Klassen in Seesedimenten mit hoher Genauigkeit erkennen und klassifizieren. Der Ansatz ermöglicht somit eine vollautomatische Analyse, wenn eine begrenzte taxonomische Auflösung der Analysen ausreichend ist. Wenn eine vollständige taxonomische Auflösung erforderlich ist, kann TOFSI in einem halbautomatischen Ansatz verwendet werden, bei dem kritische Objekte manuell überprüft werden. Beide Ansätze reduzieren die nötige Arbeitszeit erheblich, während die resultierenden Pollensummen und damit die statistische Zuverlässigkeit der Ergebnisse oft viel höher sind. Neben einer höheren Produktivität hat eine Bild‐basierte Arbeitsweise weitere Vorteile. Sie vereinfacht die Ausbildung von Studierenden und die Kommunikation zwischen Forschern. Sie erleichtert die Dokumentation und die langfristige Speicherung von Ergebnissen. Damit ermöglicht sie eine besseren Standardisierung der Pollenanalysen. Darüber hinaus ist diese Arbeitsweise schonender für die Augen.

## 1 Introduction

Almost 110 years after the first presentation of pollen diagrams (von Post 1918), pollen counts from fossil sediment records are still made by manual examination of slides, usually under a light microscope. Better microscopes, improvements in sample preparation, the availability of illustrated keys and reference collections have improved the quality of analysis, that is the number of pollen types reported has increased substantially over time. The use of keyboards or foot pedals for counting has increased the speed of analysis, but only to a limited extent. The analysis of a well‐preserved Holocene, Central European pollen sample with a pollen sum of 600 can take about 2–4 h. The analysis is slower when difficult pollen types are abundant, when preservation is poor, or when the diversity of pollen types is much higher, as in tropical regions. The slow speed of manual pollen analysis limits the use of modern, data‐intensive approaches that aim for true data‐based reconstructions of past vegetation rather than the more subjective, expert‐based interpretation of pollen records. For example, ROPES requires long pollen records with high temporal resolution (Theuerkauf and Couwenberg 2018), while the LRA (Sugita 2007) and EDA (Theuerkauf and Couwenberg 2017) require pollen data from multiple sites.

Attempts at faster, automated pollen recognition have a long history, but progress has been slow until the advent of image recognition with machine learning and deep convolutional neural networks (DCNNs) such as AlexNet since 2012 (Krizhevsky et al. 2012). A first series of applications in palynology has shown that trained DCNNs are indeed able to distinguish multiple pollen types using either ordinary bright field microscopy images (e.g., Olsson et al. 2021) or dark field light microscopy images (Sevillano et al. 2020). In these initial studies, the DCNN approach was trained and applied using images of freshly collected, that is clean and well‐preserved, pollen grains. In fossil samples from lake sediments or peat, pollen grains are instead usually less well preserved and embedded in a matrix of different remains. Models trained only on modern material therefore show poorer performance on fossil samples (Durand et al. 2024). To specifically address pollen recognition in lake sediments and peat, Theuerkauf et al. (2023) developed the two‐stage TOFSI approach. A first DCNN detects pollen, spores and other relevant objects on slide images, which are then classified by a second DCNN. Trained on images of fossil pollen samples, the approach was able to recognise 10 types of pollen and lycopodium spores on images of fossil samples with high accuracy. Recall, which reflects the proportion of all, for example alnus pollen grains present in a sample that have actually been classified as alnus, is on average 84%. Precision, which reflects the proportion of objects classified as, for example alnus that are actually alnus pollen grains, is on average 93%.

To date, two studies have demonstrated the application of automated pollen recognition to fossil pollen samples. von Allmen et al. (2024) also used a two‐step approach with object detection followed by object classification. The model was trained on images of fossil samples. In a test application it was used to automatically classify 9 pollen types and several NPPs from 26 samples of a Lateglacial pollen record from Switzerland. The results suggest an intermediate performance. While the average recall is 90%, the average precision is only 61%, meaning that most of the pollen belonging to the 9 pollen types was indeed found, but a number of other objects were also classified as one of the 9 pollen types. Durand et al. (2024) instead developed an approach with multiple DCNNs, combined in a hierarchical model. The approach has been trained to recognise 18 pollen types using images from reference collections or from freshly collected pollen. It performs well with similar images of modern pollen. However, an application on 271 fossil samples from a Holocene pollen record shows large discrepancies between the automatically created record and samples counted by a human analyst.

Here, we demonstrate application of the TOFSI approach to a 4.6 m long sediment record from the palaeolake Kieshofer Moor in Northeastern Germany. The record has been sampled continuously at very high, 1 cm resolution, resulting in 464 samples. For evaluation, a selection of samples has been revised manually. Moreover, the resulting pollen diagram is compared to a complete yet lower resolution, manually counted pollen record from the same site. We discuss the practical aspects of automatic pollen recognition, its benefits and limitations, and finally suggest a path to include automatic pollen recognition into routine pollen analysis.

## 2 Methods

### 2.1 Pollen Data

The pollen samples for automatic recognition were taken from two cores, KM23B and KM23C. Both cores were taken in January and December 2023, respectively, in the palaeolake Kieshofer Moor near Greifswald, Northeastern Germany at a distance of 0.5 m (5,412,931, 1,334,110). Cores were taken with a Swedish chamber corer (chamber = 100 cm long and 7 cm in diameter). Pollen samples were taken continuously by first pushing a U‐channel (8 × 8 mm) into the sediment and then cutting 1 cm slices. The resulting sample volume is 0.64 cm^3^. KM23B was sampled completely except for the usually disturbed top 10 cm of each core segment. From KM23C, only the middle 20/25 cm of each core segment was sampled to cover the gaps in KM23B. The KM23C samples were aligned with the KM23B samples using their pollen stratigraphy.

Pollen samples were prepared according to the standard protocol of Fægri and Iversen (1989), including treatment with 25% hydrochloric acid, 10% sodium hydroxide and acetolysis at 100°C for 7 min. Samples from 542 to 667 cm depth were additionally treated with cold 40% HF for 4 days. Prior to treatment, two Lycopodium spore tablets distributed by Lund University (batch 280,521,291 with ~13,761 spores per tablet) were added to each sample as exotic marker (Stockmarr 1971). All samples were mounted in silicone oil (viscosity = 2000 cst). Sample slides were prepared with 23 × 23 mm coverslips sealed with transparent latex paint.

An age‐depth model of the KM23B/C record was constructed using the R package Bacon v2.2 (Blaauw and Christen 2011) using four radiocarbon dates, the distinctive Laacher See tephra, and four known biostratigraphic marker horizons (Table 1). The radiocarbon dates were obtained on biostratigraphic marker horizons of the older core KM1 from the same site. They have been transferred to the equivalent biostratigraphic horizons of KM23. The mean sample resolution is 25 years in KM23 and 75 years in KM1.

**TABLE 1:** Dating points of KM23B/C and KM1.

Depth KM23B/C (cm)	Depth KM1 (cm)	Radiocarbon age (BP)	Calibrated age (cal. BP)	Source	Description
200	240		1100	Dörfler et al. (2012)	alnus‐minimum
219	248	1160 ± 30	1255	Poz‐35884	Centre of fagus peak
360	442	4300 ± 35	4874	Poz‐35887	ulmus/quercus peak
383	475		5850	Dörfler et al. (2012)	ulmus decline
432	530		8100	Dörfler et al. (2012)	fraxinus increase
460	567	8170 ± 50	9121	Poz‐35889	Begin tilia increase
520	655	8900 ± 50	10,243	Poz‐35890	Pause in corylus increase
615	790		11,600	Brauer et al. (1999)	End of Younger Dryas
640	812		12,680	Brauer et al. (1999)	Onset of Younger Dryas
654	820		12,880	Brauer et al. (1999)	Laacher See Tephra

### 2.2 Imaging

Sample slides were scanned using a modified Zeiss Axioskop 40 microscope equipped with a motorised Prior Optiscan XY stage and a motorised Prior focus drive. Images were captured using a Jenoptic Gryphax Subra microscope camera (2.3 MP = 1920 × 1200 pixel resolution) mounted on a Zeiss 60 C 2/3″ 0.63× video adapter. The pixel size of the resulting images is 0.23 × 0.23 μm. In order to detect objects at the edges of the image, images were acquired with ~10% overlap. To account for the low depth of field, a stack of 13 images was acquired at each location with ~2.5 μm distance in the z‐direction. Each set of 13 images was saved as a Tiff image container, with jpeg compression applied to each individual image. For each slide, a central area of 12 × 12 mm was scanned. In the Holocene section, this area usually contained a sufficient number of pollen. In sections with low pollen concentrations, namely the Younger Dryas, several slides were scanned per sample.

### 2.3 Automatic Pollen Recognition

For automatic pollen recognition we used the TOFSI approach for automatic recognition of pollen, spores and other microscopic objects in lake sediments (Theuerkauf et al. 2023). TOFSI consists of two stages. The ‘detector’ finds pollen, spores and other objects of interest in the sample matrix. The ‘classifier’ then classifies all detected objects into the trained categories. To estimate a confidence of the classification, the Softmax function is applied to the raw outputs of the classifier neural network. It creates a probability distribution, that is assigns a probability score to each of the different classes. A detailed description can be found in Theuerkauf et al. (2023).

The model is trained using annotated image stacks of light microscope slide scans. High accuracy in the detection and classification of fossil pollen and spores requires the model to be trained on images of fossil samples, although the preparation of the necessary training data is much more time‐consuming than when using freshly collected pollen or reference slides.

The first example application of TOFSI included 10 pollen types and lycopodium spores. For the present study, we aimed to include all common pollen and spore types of the palaeolake Kieshofer Moor pollen record, as well as selected NPPs. The number of training classes was therefore increased to 48 (Table 2). To deal with questionable objects, we introduced the classes ‘other’ and ‘nonpollen’. ‘Other’ includes all pollen and spores not belonging to any of the training classes, for instance because their counts are too low to train on. ‘Nonpollen’ includes various indistinguishable objects that are regularly present in the samples. This class allows the TOFSI classifier to filter out as irrelevant ‘nonpollen’ objects that were incorrectly selected by the TOFSI detector. Additional objects that still were clearly recognisable as pollen or spore, but too degraded or blurry to be classified accurately, were labelled as ‘undeterminable’. The classifier tries to assign a low confidence value to those objects.

**TABLE 2:** Trained classes and the number of training instances.

No.	Class	No of objects	Status
	*Arboreal pollen*		
1	acer	14	?
2	alnus	808	+++
3	betula	965	+++
4	carpinus	91	+++
5	corylus	675	+++
6	fagus	265	+++
7	fraxinus	141	(+++)
8	picea	18	?
9	picea saccus	20	?
10	pinus	579	+++
11	pinus saccus	1145	+++
12	quercus	582	+++
13	salix	219	+++
14	tilia	208	+++
15	ulmus	305	+++
	*Herbal pollen and spores*		
16	artemisia	84	+++
17	asteraceae	12	?
18	Calluna vulgaris	93	+++
19	cannabis	26	?
20	caryophyllaceae	4	?
21	*centaurea cyanus*	5	?
22	cerealia	51	?
23	chenopodiaceae	42	+
24	cyperaceae	107	+++
25	equisetum	18	?
26	ericaceae	11	+
27	filipendula	100	?
28	lactuceae	6	?
29	lycopodium (exotic marker)	589	+++
30	Plantago lanceolata	66	+
31	monolete spores without perine	103	+++
32	rumex	154	+
33	secale	231	+
34	sinapis	19	+
35	sparganium	20	?
36	sphagnum	1292	+
37	thelypteris	30	?
38	Typha latifolia	1	?
39	umbelliferae	6	?
40	wild grass group	356	+++
	*Non‐pollen palynomorphs*		
41	assulina	29	?
42	botryococcus	98	?
43	micro‐charcoal	590	+++
44	fungal spore	21	?
45	rotifera eggs	640	+
46	pediastrum	166	+
	*Additional classes*		
47	Other	2344	
48	Nonpollen	2708	

*Note:* The status column indicates performance of automatic pollen recognition: ‘+++’ high precision and recall determined in test data, ‘+’ too rare for statistical analysis in the test samples, yet matching curves in KM23 and KM1, ‘?’ too rare in the test samples and the diagrams for evaluation.

To in the text clearly distinguish between pollen, spore and NPP classes and inferred taxa, the classes are plotted in small caps.

Previous tests have shown that pollen recognition with TOFSI is sensitive to variations in image settings as well as variations in the sample matrix, that is the type and abundance of detrital remains present in a sample. For maximum robustness, the new TOFSI model was therefore trained on a variety of samples from six pollen datasets from Northeastern Germany, including samples from KM23B and KM23C itself (Table 3).

**TABLE 3:** Origin of pollen samples used to generate training data.

Site/Record	Type of deposit	No. of samples	No. of images
Lake Heiliger See (short core)	Lake sediment	10	2785
Kieshofer Moor/KM1	Lake sediment	1	50
Kieshofer Moor/KM23B	Lake sediment	5	2669
Kieshofer Moor/KM23C	Lake sediment	12	2148
Krampnitz/Long core	Peat	2	476
Tiefer See/long core the from lake centre	Lake sediment	1	99
Sum		31	8227

In order to generate training data as efficiently as possible, we used a three‐step approach. First, all images were analysed using a previous TOFSI model and cut‐out images of all detected objects were stored in respective class folders, for example alnus, betula, etc. The second step was to check all the cut‐out images. Wrongly classified images were moved to the correct folder and the annotations of each image were corrected accordingly using a Python script. In the third step, all full image stacks were reviewed using the TOFSI user interface to also annotate objects that were not detected during the initial TOFSI application. This procedure proved to be several times faster than annotating images fully manually. In addition, to ensure good representation of the rarer classes, image stacks were filtered after the initial TOFSI application, that is only stacks containing at least one object from one of the rarer classes were used as training data. The resulting training data was used to simultaneously train both the TOFSI detector and the classifier using the default settings. The resulting new model (‘Model 2024‐03‐29 Data set HGW’) was used for automatic pollen recognition of all KM23 samples.

### 2.4 Evaluation

Automatic classifications with TOFSI were evaluated in two ways. One sample every 50 cm was prepared as a test data set in two validation steps. Following TOFSI application, all detected and classified objects were written into class folders. All folders were manually checked and wrong classification were corrected. For a second check and in order to detect pollen and spores that were not detected by TOFSI, all image stacks were reviewed and corrected. For each test samples, this provides the confusion matrix of TOFSI generated and manually corrected classifications. Using these test data, the class‐specific precision and recall of the automatic classification was calculated. Precision for each class describes what proportion of all objects classified as, for example alnus pollen, has been classified correctly, that is are actually alnus pollen grains. Precision is calculated as the ratio of true positives to the total number of detected pollen (which is equal to the sum of true positives and false positives). Conversely, the recall for each class characterises what proportion of all, for example alnus pollen present in a sample has been successfully detected by the algorithm. Recall is calculated by dividing the number of true positives by the total number of annotated pollen (which equals the sum of true positives and false negatives). Mathematically this is expressed as:

$$\text{Precision}=\frac{\text{truepositives}}{\text{totaldetected}},\text{recall}=\frac{\text{truepositives}}{\text{totalannotated}}$$

In order to assess, which confidence level is most appropriate for reporting results, we calculated precision and recall at different confidence levels.

As additional performance indicators we determined the F1 score and Matthews correlation coefficient (MCC). The F1 score is the harmonic mean of precision and recall, expressed as:

$$F1=2\times \frac{\text{precision}\times \text{recall}}{\text{precision}+\text{recall}}$$

The MCC score was calculated using the ‘matthews_corrcoef’ function from the python package ‘sklearn.metrics’. Calculations were for the entirety of the test samples. Only the well trained classes (+++ in Table 1) are included in the analysis, except for micro‐charcoal, as the focus is on pollen and spores here.

All performance parameters were calculated so that at a given confidence level, all classifications that are correct but at a lower confidence level are considered as incorrect, that is as false negatives. Confidence intervals (95%) for all metrics were estimated using bootstrap resampling (*n* = 1000 iterations), whereby the complete dataset including undetected objects was resampled with replacement at each confidence threshold.

Besides classification statistics, the automatically counted pollen record KM23 is compared with the manually counted pollen record KM1 from the same site. KM1 was already cored in 2007. The sequence is overall 1 m longer than KM23, yet covers the same time period, starting shortly before the Laacher See eruption. KM1 was mostly sampled at 2.5 cm intervals, that is the sample resolution is lower than in KM23. Pollen samples were prepared as in KM23. Samples were analysed manually at 400× magnification using a Zeiss Axioskop 40 microscope. Pollen percentages of KM1 and KM23 were calculated using the same upland pollen sum. Because of the later mentioned error in the recognition of fraxinus, this pollen type was excluded from the pollen sum in both records. An age‐depth model for KM23B/C was created using the R package Bacon v2.2 (Blaauw and Christen 2011) using the same dating points as for KM1 (Table 1).

## 3 Results

### 3.1 Evaluation of TOFSI Performance in the Test Samples

**FIGURE 1:**
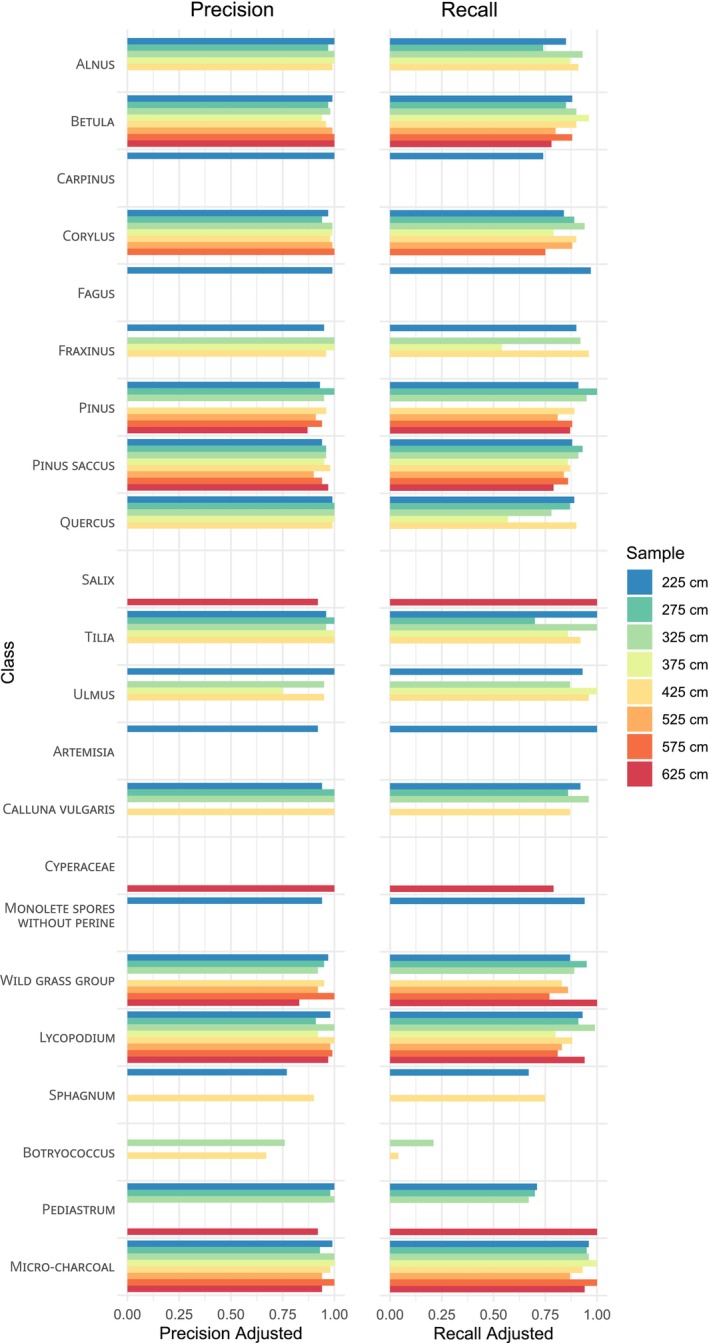
Precision and recall of the automatic recognition at the 0.5 confidence level. Precision and recall are only shown for classes and samples with a minimum of 10 objects present.

In the eight test samples, and considering all objects classified with a confidence greater than 0.5, the precision for all recurrent classes is mostly well above 0.9 (Figure 1), meaning that more than 90% of the classifications made by TOFSI are correct. Precision is only lower for sphagnum (~0.8) and botryococcus (~0.7). At the same confidence level, recall is well above 0.8 for the major tree and herb pollen types and the exotic lycopodium spore marker, meaning that for these classes, TOFSI detected and correctly classified more than 80% of the pollen/spores present in the test sample images. The undetected objects are often, but not always, those obscured by detritus or other pollen and spores, as discussed below. For botryococcus, recall is exceptionally low (< 0.25). Obviously, the current TOFSI model is not suitable for detecting these algal colonies, although the higher precision (~0.7) shows that most of the objects classified as botryococcus were indeed botryococcus.

The present results show little variation in precision and recall between the test samples, except for sample 375 cm. Here recall is exceptionally low for fraxinus and quercus and precision is exceptionally low for ulmus. In total only 427 pollen and spores were detected in this sample, mainly because pollen and spores are difficult to identify as they are contained in detrital aggregates.

**FIGURE 2:**
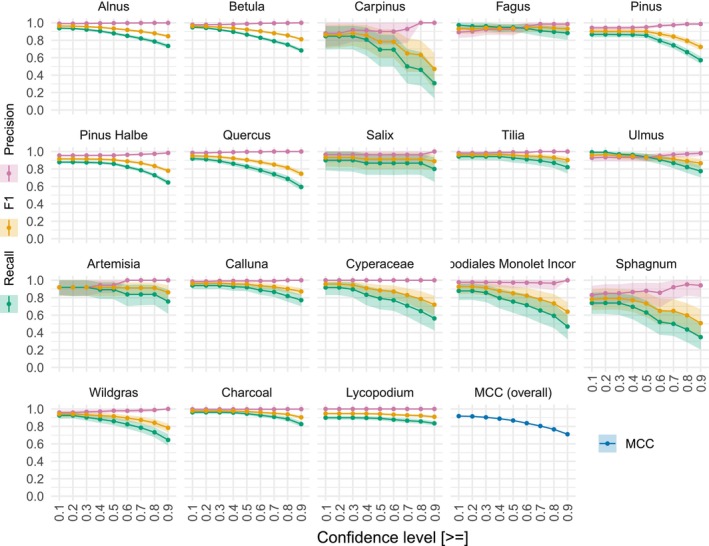
Precision, recall and F1 score and their 95% confidence intervals calculated for automatic classifications with increasing confidence levels, for all well trained classes and summarised over all test samples. Additionally, the bottom right panel shows the MCC score (including 95% confidence interval) over all test samples.

**FIGURE 3:**
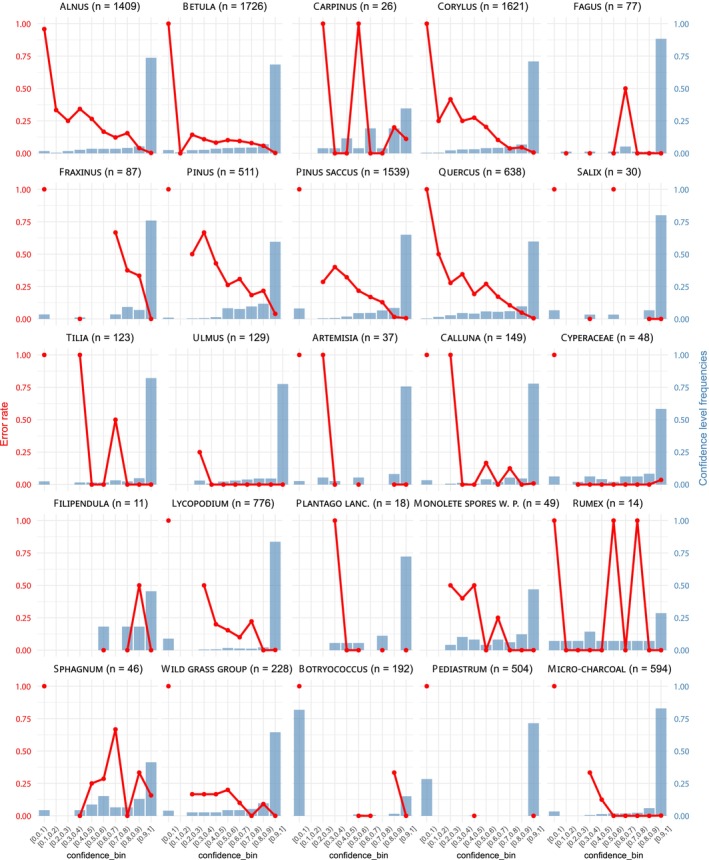
The distribution of confidence values assigned by TOFSI for all objects of each class in steps of 0.1 (blue bars). A confidence of 0 represents objects that were not detect by TOFSI. For each confidence level, the error rate, that is the ratio of the number of correctly classified objects and the total number of objects, is shown in red dots.

As expected, the precision of automatic recognition is highest when only classifications with high confidence are considered, reflecting that errors tend to occur more often for objects classified with low confidence (Figure 2). Recall, on the other hand, is lowest when only classifications with high confidence are considered, because low confidence classifications are also correct to some extent. In the present dataset, lowering the confidence level from, for example 0.9–0.5 has a large effect on recall, which increases considerably, but a small effect on precision, which decreases only slightly (Figure 2). This pattern reflects a rather high proportion of correct classifications even at intermediate confidence levels. For the common tree and herb pollen classes, as well as for lycopodium and micro‐charcoal, the error rates for intermediate confidence classifications are around 25%, that is 75% of the classifications are correct at a confidence level of ~0.5 (Figure 3). Moreover, for these common classes, around 75% of all objects present have been classified with the highest confidence. The error rate for the highest confidence level tends to be zero, that is almost all of these classifications are correct. The error rates are more variable for less well trained classes.

F1 scores, the harmonic mean of recall and precision, are mostly above 0.8 (Figure 2). Such high values are commonly considered as indicating good model performance, confirming the mostly good performance visible in precision and recall. F1 scores decline at higher confidence levels, following the trend in recall, suggesting that the higher recall at lower and intermediate confidence levels outweighs the higher precision at high confidence levels. The MCC is considered a more robust measure of quality, namely in imbalanced data sets. MCC is mostly high (> 0.8), except for the highest confidence levels, also confirming the good performance of the TOFSI approach (Figure 2).

**FIGURE 4:**
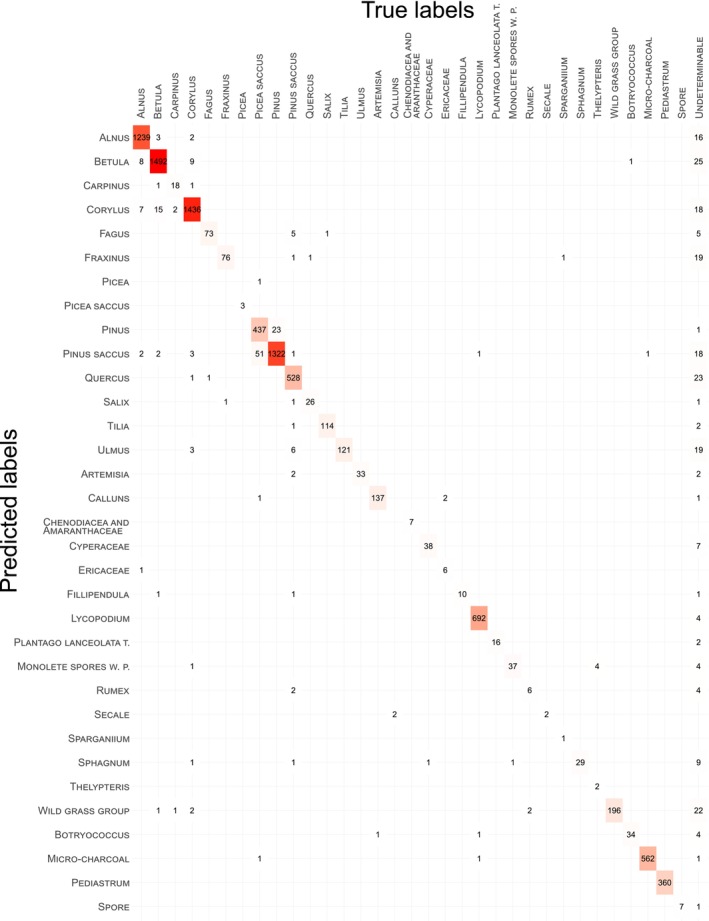
Confusion matrix comparing the number of true and predicted objects present for each class, calculated including all classifications with > 0.5 confidence.

**FIGURE 5:**
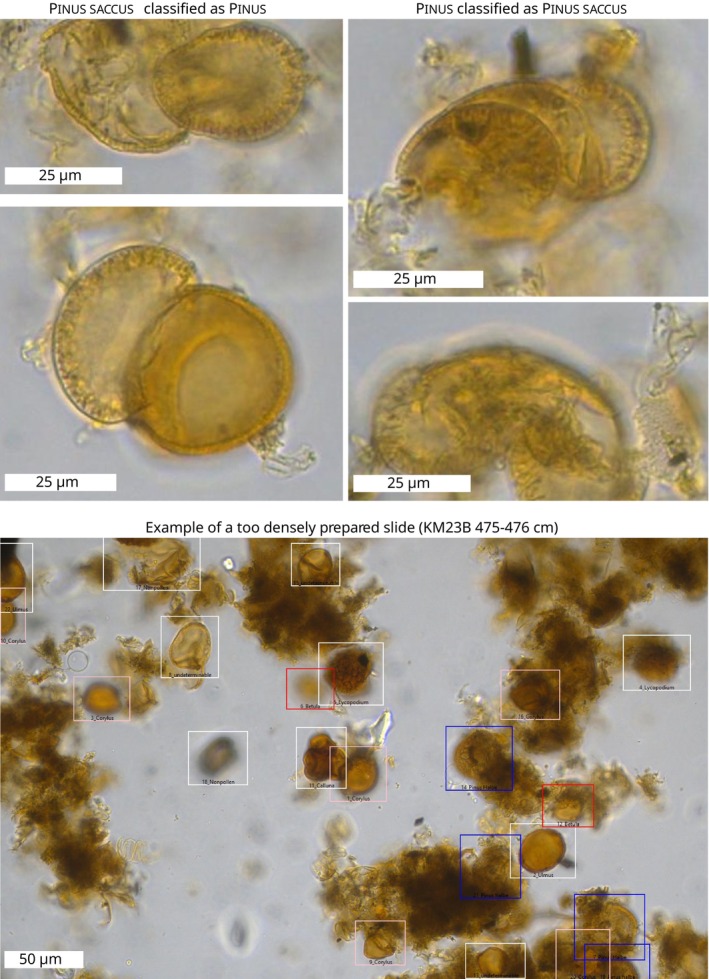
Above examples of incomplete Pinus pollen grains with only one saccus (pinus saccus) that were wrongly classified as complete Pinus pollen grains with two sacci (pinus) and vice versa. Below an example of a too densely prepared slide with aggregates of detrital matter. Nonetheless, most pollen grains are still detected and classified correctly.

TOFSI regularly, even though still rarely, confuses the similar pollen types betula and corylus, and to lesser degree alnus (Figure 4). About 1% of all betula pollen has been wrongly classified as corylus, and about 0.6% of all corylus pollen as betula. Of all alnus pollen, also around 0.6% have been wrongly classified as either betula or corylus, but only about 0.2% of all betula and corylus pollen grains have been wrongly classified as alnus. Larger errors occur for complete pinus pollen grains and pinus saccus. About 10% of all complete pinus grains has been classified as single pinus saccus, yet less than 2% of pinus saccus as complete pinus grains. These errors likely mostly relate to grains that are folded, visible in some side‐view or located at the image margins (Figure 5). In some rare cases, also alnus, betula and corylus pollen were wrongly classified as pinus saccus. Moreover, about 4% of all the quercus pollen grains (*N* = 549) were wrongly classified, most of them as ulmus (1%) and fagus (0.9%). All wild grass group pollen grains were classified as such, however in rare cases also betula, carpinus, corylus and rumex pollen grains were classified as wild grass group pollen. Errors are remarkably small in the case of lycopodium as exotic marker. Only 3 of 695 spores (0.4%) were wrongly classified.

### 3.2 Comparison of the KM23 and the KM1 Pollen Records

**FIGURE 6:**
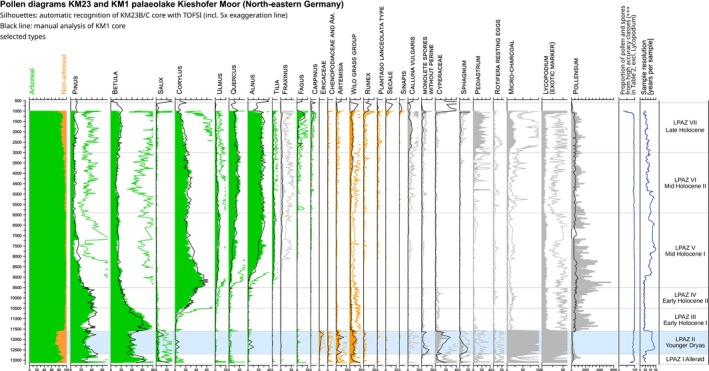
Automatically counted pollen diagram KM23 (silhouettes with 5× exaggeration line) and manually counted pollen diagram KM1 (black lines). The pollen types included in the pollen sum are indicated in green (trees) and orange (herbs).

The good performance of TOFSI in the eight manually reviewed test samples is visually confirmed by the close match between the automatically generated composite pollen record KM23 and the manually counted pollen record KM1 (Figure 6). Both pollen records show largely similar values, with only some minor differences.

During the Early Holocene (11,600–10,500 cal. BP), pinus pollen percentages are slightly higher in KM1, while betula pollen percentages are slightly higher in KM23. It is unlikely that these differences reflect errors in the automatic counts, since the two pollen types appear distinctly different and were therefore very rarely confused in the test samples. Instead, we assume that there are real differences between the two records. Both sequences were taken at a distance of ~50 m from each other, that is they were probably at different distances from the lake shore at the time. For pinus, pollen deposition can vary considerably across a lake basin. As this pollen type floats for longer, it may be preferentially deposited on the windward side of a lake (Ammann 1994; de Klerk and Hölzer 2009). Due to high extra‐local pollen deposition from birch trees growing on the lake shore, betula values may be higher closer to the shore. From 7000 to 8500 cal. BP, quercus and alnus pollen percentages tend to be slightly higher and pinus pollen percentages slightly lower in KM23. Again, it seems unlikely that pinus pollen grains were misclassified as alnus or quercus, as no such errors occurred in the test samples. As above, we expect that the differences between the two pollen records reflect true differences in pollen deposition at the two sites. Around 5700 cal. BP, KM23 shows a short‐lived peak in fagus that is not present in KM1, obviously due to its lower sampling resolution. The most striking difference between KM23 and KM1 is in the top partof the record, during the terrestrialisation of the palaeolake. At this level, KM23 shows a prominent peak in fraxinus pollen percentages that is not present in KM1. Manual revision shows that this mismatch reflects a true classification error—the abundant sparganium pollen at this level was partly misclassified as fraxinus by TOFSI.

## 4 Discussion

### 4.1 Automatic Pollen Recognition With Fossil Pollen Samples

Although previous studies have demonstrated the effectiveness of machine learning approaches in accurately classifying multiple pollen types in fresh reference material (e.g., Olsson et al. 2021; Sevillano et al. 2020), detecting and identifying pollen in fossil samples is substantially more difficult. In this study, we demonstrate that the TOFSI approach can effectively and accurately identify pollen, spores and NPPs in fossil lake sediment samples. Our test samples prove the accurate identification of 12 tree pollen classes, six herb pollen/spore classes, and two NPP classes. The good performance for these more abundant classes is shown in various scores, that is precision, recall, F1, and MCC. For further, rarer classes, the high degree of similarity between the manually counted pollen record KM1 and the automatically counted pollen record KM23 suggests that all recurrent pollen, spore and NPP classes (27) were in fact detected and classified with high accuracy, except for fraxinus and sparganium. Ongoing tests with the current TOFSI software suggest that the number of detectable classes can well be increased further.

Durand et al. (2024) found that models trained using modern reference material performed well with this material but not with fossil samples. Like von Allmen et al. (2024), the present study shows that models perform better with fossil samples when they were also trained using fossil sample material. Although labelling such images is tedious, it is necessary for the model to learn to distinguish between target objects (pollen, spores and NPPs) and other objects (detritus, etc.). The training images from fossil samples also include various stages of degradation of the objects. Unlike humans, who focus on morphological characteristics such as shape, aperture type, or pore structure, artificial neural networks primarily rely on pixel‐level features such as texture, edges, and local contrast. This makes them sensitive to variations in preservation state, previously unseen objects such as dirt or image acquisition parameters. In order to recognise objects successfully, the models need to be trained using a large number of objects with a wide variety of appearances, including a variety of backgrounds. While modern reference material can easily provide a large quantity of objects, it cannot provide the necessary variety in pollen and image backgrounds.

Our TOFSI model, like the one of von Allmen et al. (2024), has a two‐step design involving the separate detection and classification of target objects. The good performance of both models when working with fossil samples suggests that this design is beneficial in this context—a separate detector can rapidly locate the often scarce pollen, spores and NPPs within the matrix of detrital remains. To enable fast processing, the detector in the TOFSI model is used with reduced image resolution. Even crowded images are usually processed in less than 1 s. Surprisingly, the detector reliably detects not only pollen from the trained classes, but also most pollen from classes not yet included in the training data (Theuerkauf et al. 2023). In many cases, these unknown pollen types are classified as ‘Others’ by the TOFSI classifier, though some are incorrectly assigned to one of the trained classes. As we will discuss below, the detector's ability to identify unknown pollen is potentially useful for establishing a hybrid workflow.

Despite the high level of accuracy overall, our test data show some variation in the magnitude of errors made in pollen classification. Unmistakable classes, such as tilia and lycopodium, were rarely misclassified. In some cases, lycopodium spores remained undetected, likely due to their differing appearance in polar and equatorial view. Misclassifications occurred somewhat more often among groups of similar pollen types, such as alnus, betula and corylus, albeit in fewer than 1% of cases. Errors of this magnitude are negligible when all pollen types are present in significant quantities, that is during most of the Holocene. They may be problematic in periods when only one of the corresponding tree taxa was actually present, like birch during colder, (Late)glacial periods. In such cases, classification errors may incorrectly suggest the presence of the warm loving tree taxa alder and hazel during cold periods. We found that both alnus and corylus pollen is indeed present in Lateglacial sections of KM23 and KM1—likely originating from older, reworked material.

The largest error was that sparganium pollen was to some extend misclassified as fraxinus. Consequently, the fraxinus peak in PAZ8 of KM23 is mostly a sparganium peak, which reflects the terrestrialisation of the palaeolake Kieshofer Moor with, for example *Sparganium* or *Typha angustifolia* reeds. sparganium pollen is rare in other parts of the record, so misclassifications as fraxinus are most relevant in the uppermost section. fraxinus and sparganium pollen grains have a similar micro‐reticulate surface pattern and are therefore sometimes mistaken for each other by inexperienced human analysts. However, fraxinus pollen is tricolpate, whereas sparganium pollen is monoporate. We assume that the misclassifications result from sparganium being under‐represented in the training data (*n* = 20). Future tests will reveal whether additional training data mitigates the issue, or whether the misclassifications indicate an inherent limitation of the approach when dealing with similar pollen types.

### 4.2 Data Selection

In our TOFSI approach, a confidence level is estimated for each classification. For the KM23 diagram, we have chosen to include all classifications with a confidence level above 0.5 because error rates remain low at this level (approximately 25%), particularly for the main pollen types. Setting the threshold at 0.5 therefore increases the recall while maintaining high precision. For pollen types that are already well trained and abundant, an even lower threshold would be acceptable because error rates remain low in low‐confidence classes; that is to say, more correct than incorrect classifications will be included. For less well trained classes, which usually have higher error rates in lower confidence classes, a too‐low threshold would be problematic. Therefore, the optimal confidence threshold remains a matter of debate and requires test statistics to be estimated.

Even without a test statistic, the distribution of confidence values for each class indicates the model's performance. A high proportion of high‐confidence classifications (e.g., 75% of classifications with a confidence ≥ 0.9, as for alnus, fagus and tilia) indicates good performance. Conversely, a high proportion of low confidence classifications indicates poorer performance and the need for additional training, as seen with carpinus, rumex and sphagnum.

### 4.3 Benefits, Limitations and Potential Application

TOFSI‐based automatic analysis is much faster than manual pollen analysis. Our workflow involves setting up the microscope for slide scanning and managing image and data files, as well as applying the TOFSI approach. With the current microscope setup, we can scan four slides, each with three samples (with 23 × 23 mm cover glasses) in a row. In most cases, scanning a central area measuring 14 × 14 mm, which takes approximately 2 h, is sufficient. Hence, up to 12 samples can be scanned per day. The TOFSI application takes between 30 and 60 min to complete on a mid‐range personal computer with a mid‐range graphics card. Overall, our workflow enables us to process up to 12 samples per day, with sample preparation and scanning being the main limitations. Manual tasks, such as setting up the microscope and managing files, take less than an hour in total for each daily round—less than 5 min per sample. By contrast, manually analysing KM1 samples with a target pollen sum of 600 upland pollen takes at least 2 h per sample—at least 12 times longer. Moreover, in many cases, the pollen sum of samples analysed automatically is much higher, providing greater statistical reliability of the pollen percentages.

The key limitation of the automatic recognition method presented here is its limited taxonomic resolution. As discussed above, we opted to train TOFSI using images of fossil pollen samples in order to achieve the best possible results. However, manually labelling thousands of images is time‐consuming, particularly for rare pollen types. Consequently, namely many herbal pollen types are under‐represented or entirely absent from our current training dataset. In addition, properties of the microscope images taken with 40× objective lenses also limits taxonomic resolution. The identification of many, namely herbal pollen types requires either higher resolution (i.e., 100× oil objective lenses) and/or phase contrast. Scanning at higher resolution is possible with our setup, yet at least 10 times slower. It also produces mostly unnecessary large amounts of image data.

Considering the benefits and limitations of automatic pollen recognition with TOFSI, we propose three potential applications that involve different degrees of manual correction, and therefore result in different levels of accuracy and taxonomic resolution.

#### 4.3.1 Fully Automatic—No Correction

The KM23 record demonstrates that fully automated analysis can produce high‐resolution pollen records with high accuracy yet limited taxonomic resolution. These records are obviously useful when high temporal resolution is more important than taxonomic resolution. This may include studies of basic vegetation reconstruction, biostratigraphic dating or the reconstruction of past masting years using annually laminated lake sediments (Theuerkauf et al. 2019). Despite the mostly high accuracy observed here, errors like with fraxinus and sparganium show that some manual revision of major types is advisable.

#### 4.3.2 Cutout‐Correction

When higher reliability and taxonomic resolution are required, automatic recognition can be supplemented by manually verifying all detected objects. To this end, cut‐out images of each object are stored in separate class folders, ordered by the assigned confidence level. These cut‐outs retain all image layers for the most accurate revision possible. Depending on the overall number of objects present, their preservation, the amount of detrital background and the abundance of difficult pollen and spores, going through these folders using, for example miniature view in Windows Explorer, can be very fast. For samples with well‐preserved pollen and spores, not too many difficult types, and for a pollen sum of ~1000, such revision can be done in 10–20 min, that is much faster than full manual analysis under a microscope. One limitation of this approach is that it only covers the pollen, spores, etc. that TOFSI has indeed detected. However, even when trained on only 10 pollen and spore classes, TOFSI detected more than 95% of pollen from the trained classes and surprisingly also around 90% of pollen from the yet untrained classes (Theuerkauf et al. 2023). This means that very few pollen grains remain undetected. When more types are included in the training data, the already low number of undetected pollen grains is likely to decrease further. Ideally, TOFSI would classify unknown pollen as ‘others’, or in some cases as one of the trained classes. In the present application, objects in the ‘others’ class can be manually classified, and errors can be corrected.

#### 4.3.3 Hybrid Approach

In some cases, image cut‐outs are not suitable for robust classification, for example because pollen grains are degraded or hidden, or features are invisible. When a complete analysis of all pollen and spores in a sample is required (e.g., for biodiversity research), a hybrid approach can be used, whereby critical grains are examined under a microscope. We scanned all samples using an ordinary light microscope that is additionally equipped with a motorised stage and focus drive, and on classical slides. This setup allows both scanning of samples and ordinary manual pollen analysis. In our workflow, the coordinates of each image and each detected object within it are preserved. Using suitable software tools, slides can be automatically placed at the location of critical objects for manual revision under the microscope if needed, for example with oil immersion for higher resolution or phase contrast. Depending on the mounting medium, objects may be moved for better visibility. This approach combines the high speed of automatic recognition of well‐detectable objects with expert classification of critical pollen and spores. Although we have not yet implemented this approach, we assume that it allows for substantially faster analysis than a complete manual approach. The additional benefits of working with digital images are discussed below. As for the other approaches, pollen grains that remain undetected by TOFSI will be missed in this analysis, yet this limitation is likely small. Nonetheless, we in any case recommend also the revision of the full scanned images in either regular distances or for distinct levels to estimate complete error rates.

#### 4.3.4 Benefits and Limitations of a Digital Workflow

The use of automatic recognition approaches not only has the potential to provide much faster analyses. It also implies a shift from a microscope‐based to a digital image‐based workflow, with a number of potential benefits. From our current experience, one such benefit is that pollen analysis could become a less solitary, more collaborative activity. Digital images can easily be shared and discussed with colleagues, both within labs and remotely. Such image sessions can be highly productive and enjoyable. Image sessions are also very useful for teaching, as they facilitate group discussions and enable more accurate evaluation of student capabilities than microscope count sheets.

Moreover, palynologists are increasingly sharing their raw pollen counts in databases such as NEOTOMA and the EPD. One issue is that the definition of recognised pollen types varies between labs and researchers due to different traditions, determination keys, and reference collections. Such terminological issues limit, for example meta analysis for biodiversity studies or detailed studies on particular pollen types (de Klerk et al. 2015). With the digital workflow, images of all counted objects are available. These images can also be uploaded to pollen databases, allowing for later reanalysis and verification of the classification. Therefore, image‐based approaches may not only enable more collaborative but also more objective and harmonised analysis. Even though long‐term storage of digital data has its problems, the slide scans themselves are potentially useful as a sample archive. The scans can be analysed again later using newer generation models to achieve more accurate results and detect further classes, including non‐pollen types. Another benefit of a digital workflow is that it enables various parameters of the found objects to be determined. The size parameters of birch pollen, for example are valuable for refining their classification (Theuerkauf, Nehring, et al. 2024). Similarly, size parameters of micro‐charcoal particles can be determined to estimate the overall amount of micro‐charcoal present in a sample far more accurately than by counting them in size classes. Finally, from personal experience, working primarily on a large computer screen is more eye‐friendly than long microscope sessions, certainly in an advanced age.

We experienced that in some cases, the image quality is insufficient for evaluating critical pollen features. To reduce this limitation, the scanning system should be set up so that the camera resolution is high enough to capture all visible pollen features at a given optical resolution. Images will usually not be available at the highest possible optical resolution (with 100× magnification and oil objectives) because, like routine analysis, scanning with the highest resolution is very slow. As previously mentioned, we generally recommend scanning slides with motorised light microscopes rather than ‘black box’ slide scanners because they enable critical objects to be revisited manually at higher resolution and with phase contrast. Such a system can be configured so that after a first application of automatic recognition, sample regions containing specific pollen types (e.g., Cerealia) are scanned again at a higher resolution, as in the proposed hybrid approach. Also pollen and spores that are hidden or located in an unfavourable position can be revisited again after moving them for better visibility by applying pressure to the cover glass with a needle. To minimise these issues in the digital workflow, slides should be prepared using sufficiently diluted sample material to minimise overlap (Figure 5). Sample should be prepared as thin as possible. The number of z‐stack images and their spacing should be adjusted to ensure that all parts of the pollen grains can be examined.

#### 4.3.5 Vegetation History

Pollen analysis has a long history in the study region. The first pollen diagram, also from the palaeolake Kieshofer Moor, was produced about 100 years ago by von Bülow (1928, Figure 7). Since then, multiple pollen records have been produced in the region and have enabled the postglacial vegetation history to be reconstructed in great detail (Theuerkauf, de Klerk, and Michaelis 2024). The new continuous, high resolution diagram KM23 adds valuable details. One example is the short but prominent fagus peak shortly after the elm decline. The main expansion of beech in northern Germany has been dated to around 3000–2500 cal. BP. Single earlier finds have repeatedly been reported, yet whether these reflect a much earlier presence of beech in the region or long‐distance transport events remains a matter of debate (e.g., Bradley et al. 2013). The early fagus peak in KM23 strongly suggests that beech was indeed present in the vicinity of the study site shortly after the elm decline—it may have established in the forest openings created by the elm decline. We thus hypothesise that beech was present in the area long before its main expansion, but only as small, temporary stands on disturbed sites. Automatic analysis will help to prove this hypothesis by high resolution analysis of further sites. Moreover, with automatic analysis very high counts sums are more feasible. Higher sums reduce the uncertainty in pollen data (Mosimann 1965) and hence allow to study spatial and temporal variability namely of rare pollen types with higher accuracy. Long distance transport events will create a different spatial pattern, with values increasing towards the source area, than small local populations.

**FIGURE 7:**
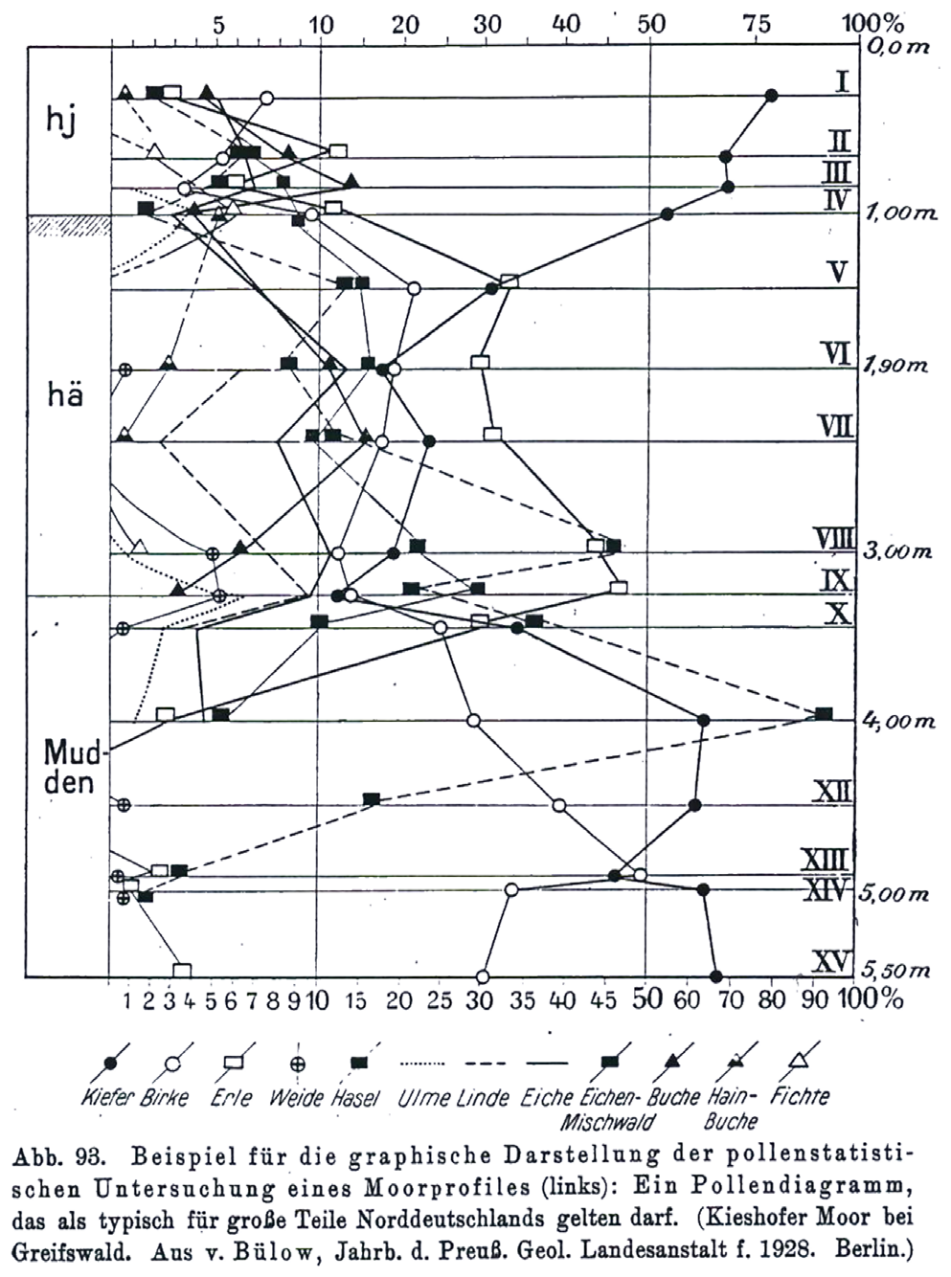
The first pollen diagram from the palaeolake Kieshofer Moor, from von Bülow (1928). The exact coring location within the peatland is unknown.

KM23 also reveals hitherto unknown patterns in pollen values of warm‐loving tree taxa during the early Holocene, such as a short‐lived decline in corylus shortly before 10,000 cal. BP and a synchronous early peak in ulmus, quercus, alnus and tilia shortly after 10,000 cal. BP. Moreover, we find indications of a response of vegetation and the lake ecosystem to the 8.2 cold event, which are hither rarely reported from the region. With similar analysis from further sites, such observations may help to better understand the postglacial tree expansion, for example in relation to high resolution climate proxies. Overall, the present example illustrates that automatic analysis has the potential to substantially refine palaeoecological research even in well studied regions.

#### 4.3.6 Outlook

The present pollen record KM23 demonstrates that the TOFSI approach enables full or semi‐automatic pollen analysis in a useful and efficient manner. The greatest limitation to date is the limited number of pollen and spore types recognised (25), due to the time‐consuming preparation of training data from fossil pollen samples. With pre‐classified images and better software tools, labelling has now become much faster and can focus on rare types.

All images used for training, testing and sample data in this study were taken with the same equipment and settings. All samples were prepared in the same laboratory using the same protocol. The performance of TOFSI can decline significantly when samples are prepared using different protocols (e.g., 2 min instead of 7 min of acetolysis) or when images are captured using different equipment. Performance may also decline with samples from different sediment types. Hence, the application of TOFSI with such differing datasets requires retraining with suitable training data. It still needs to be evaluated whether it is useful to combine all training data in order to produce one robust model suitable for all set‐ups, or to develop separate models for, for example each camera type or sample preparation protocol.

TOFSI is based on two DCNNs: Faster R‐CNN and MobileNetV3, both of which were published over 5 years ago. Preliminary tests with newer generation models have not yet shown significant performance improvements. However, given the rapid development in this field, it will certainly be useful to update TOFSI in the future.

Finally, sample preparation has not usually been a limiting factor in pollen analysis so far. However, with fast scanning and automatic recognition, it could become a limiting factor. To fully exploit the benefits of automatic pollen recognition, faster sample preparation will be required (e.g., O'Keefe and Wymer 2017; Urban et al. 2018; Durand et al. 2024).

## 5 Conclusions

The pollen record from the palaeolake Kieshof Moor demonstrates the potential of TOFSI, a DCNN‐based tool, for automatic pollen recognition in fossil samples. So far, the approach has demonstrated high accuracy in recognising common and distinct pollen and spore types, as well as selected NPPs. With additional training, recognition can likely be expanded to further classes. We currently suggest three use cases. Suitable training provided, the approach can be used without manual correction when a high sample output is more important than taxonomic resolution, for example for biostratigraphic zonation, forest history research and masting year reconstructions. For higher taxonomic resolution, all detected objects can be manually verified using image cut‐outs. Finally, to achieve the highest possible taxonomic resolution and accuracy, a hybrid approach can be adopted, whereby all critical objects are manually revised, using higher resolution and phase contrast if necessary. Sample analysis is substantially faster than full manual analysis with all three use cases, allowing vegetation reconstruction from single pollen records to be refined by, for example, increasing sample resolution and count sums. It also enables the more widespread application of data‐intensive approaches in quantitative vegetation reconstruction, such as ROPES, LRA and EDA.

Furthermore, an image‐based workflow could enhance collaboration among palynologists, facilitate training and documentation, and promote the standardisation of pollen classification.

## Author Contributions

**Martin Theuerkauf:** conceptualization (lead), data curation (lead), formal analysis (lead), methodology (supporting), software (supporting), visualization (equal), writing – original draft (lead), writing – review and editing (equal). **Alexander Gillert:** methodology (equal), software (lead), writing – review and editing (equal).

## Conflicts of Interest

The authors declare no conflicts of interest.

## Supporting information


**Data S1:** Description of the automatically counted pollen diagram KM23.

## Data Availability

The pollen counts of the two pollen records from the Kieshofer Moor palaeolake are available from the Pangaea data publisher (Felden et al. 2023) using the following links: https://doi.pangaea.de/10.1594/PANGAEA.993354 and https://doi.pangaea.de/10.1594/PANGAEA.993357. The TOFSI user interface, which allows to perform all steps from image labelling to model training and application, is available through the original application (Theuerkauf et al. 2023).
