# Supplementary material for: Efficient Automatic Pollen Recognition From Fossil Pollen Samples: A High‐Resolution Example Record From Palaeolake Kieshofer Moor, Northeastern Germany
Source: Ecol Evol. 2026 Jun 21;16(6):e73866. doi: 10.1002/ece3.73866 (PMC13284292; doi:10.1002/ece3.73866)
Supplement: Supplementary file 1 — Data S1: Description of the automatically counted pollen diagram KM23. [file ECE3-16-e73866-s001.pdf]

Supplementary material

Description of the automatically counted pollen diagram KM23

The following supplementary material describes in more detail the automatically counted pollen record KM23. To this end, the complete pollen diagram KM23 (Fig. S1) is included, as well as the manually counted pollen diagram KM1 (Fig. S2) and age-depth models (Fig. S3) of both records. For the methodology, see the main text. The age-depth model of both records is based on a rather small number of dating points, which likely implies some uncertainties. Therefore, the diagrams are here plotted and described primarily on their depth-scale. To clearly distinguish pollen types from inferred plan taxa, pollen types are written in SMALL CAPS (Joosten and de Klerk 2002).

LPAZ I – Allerød (668 – 640 cm / ~13,200 – 12,700 cal. BP)
The organogenic sediments of the KM23 start at a core depth of 668 cm, above calcareous loam. The lowermost 3 cm of the organic sediments are mixed, with a thin peaty layer recognisable at 665-666 cm. The pollen concentration in this section is very low. High pollen percentages of CYPERACEAE (up to 100 %) and SPHAGNUM (up to 30 %) suggest that this section has been deposited during an initial peatland phase, i.e. before the palaeolake formed likely due to dead ice melting. High pollen percentages of PINUS and low percentages of BETULA may indicate that this section was deposited during a very early phase of the Weichselian Lateglacial (de Klerk et al. 2008).

Above 665 cm, in detrital gyttja, the pollen concentration is much higher, and the pollen record is dominated by tree (BETULA and PINUS) and shrub (SALIX) pollen. This section represents the Lateglacial *Betula-Pinus* forest phase sensu de Klerk (2002), corresponding to the Allerød period. Elevated pollen percentages of WILD GRASS GROUP and ARTEMISIA indicate that forest in the study region were either rather open or that a pattern of dense forests and open patches existed. At 654-655, the unmistakable Laacher See Tephra is present (~12880 cal. BP, Brauer et al. 1999). Slightly below the Laacher See Tephra, pollen percentages of PINUS start to increase at the expense of BETULA, reflecting the Lateglacial expansion of pine in the study region. In central and southern Germany, this expansion started already 13,500-14,000 cal. BP, i.e. 500-1000 years earlier than in the present study region. Any vegetation response to the volcanic eruption is not detectable at the present, 1 cm, sample resolution. In PAZ1, TOFSI has repeatedly detected pollen of warm loving tree taxa, namely ALNUS, CORYLUS and QUERCUS. These finds have been confirmed by manual inspection. They most likely are older, reworked pollen grains.

LPAZ II – Younger Dryas (640 – 615 cm / 12,700-11,600 cal. BP)
Pollen-stratigraphically, the Younger Dryas period is characterised by higher percentages of herbal pollen types, namely WILD GRASS GROUP, ARTEMISIA, CHENOPODIACEAE AND AMARANTHACEAE, ERICACEAE and CYPERACEAE, and correspondingly lower tree pollen percentages. The beginning of the Younger Dryas is not sharply defined – pollen percentages of WILD GRASS GROUP pollen increase already at 650 cm, pollen percentages of ARTEMISIA at 640 cm and pollen percentages of ERICACEAE at ~630 cm. We assume that the onset of the Younger Dryas is represented by the prominent increase in ARTEMISIA at 640 cm, which also corresponds to a prominent increase in the LYCOPODIUM percentages, which reflect a decline in pollen concentrations, and the transition from an organic to a mineral rich gyttja. The end of the Younger Dryas is more sharply defined by the rapid increase in BETULA, the corresponding decline in most herb pollen types, and a rapid increase in pollen concentrations at 615 cm depth. Regionally, higher proportions of herbal pollen during the Younger Dryas have been interpreted as the return to a largely open, treeless vegetation (de Klerk 2008, Theuerkauf et al. 2014). Automatic size measurements show a shift towards smaller birch pollen during the Younger Dryas period, likely indicating an increasing proportion of dwarf birch, *Betula nana,* during the Younger Dryas (Theuerkauf et al. 2024).

Throughout LPAZ II, both manual and automatic analysis show elevated pollen values from the warm-loving tree taxa ALNUS, CORYLUS and QUERCUS. These finds are likely to be reworked material, reflecting unstable soils and the erosion of older sediments.

LPAZ III – Early Holocene I (615 – 548 cm / 11,700 – 10,500 cal. BP)
LPAZ III is characterised by an initial rapid increase in BETULA pollen percentages to a maximum of ~80 %, followed by an increase in PINUS pollen percentages to a maximum of ~45 %. Herbal pollen percentages are much lower than before, with the sum of NAP UPLAND pollen now being at around 5 %. These changes reflect the rapid reforestation of the study region after the onset of the Holocene.

In the lowermost samples of LPAZ III (608-611 cm), automatic analysis shows pollen from the warm-loving tree taxa, i.e. ALNUS, CORYLUS and QUERCUS. Like during the previous Younger Dryas, they likely originate from reworked material, pointing at ongoing erosion of older sediments during the beginning of the Holocene. Accordingly, the sediments in this section are also still rich in minerogenic material.

Even though during LPAZ III the proportion of NAP upland pollen is much lower during than during the previous Younger Dryas, several herb pollen types are still well present, namely WILD GRASS GROUP (mean = 3.9 %), ARTEMISIA (mean = 0.3 %) and FILIPENDULA (mean = 0.9 %). The percentages of WILD GRASS GROUP and FILIPENDULA even show a short-lived peak at the LPAZ II – LPAZ III transition, suggesting that they may have expanded in the warmer climate of the beginning Holocene before the expansion of trees and shrubs. Alternative, the peaks may reflect an increased pollen productivity of both taxa in the warmer climate.

Pollen percentages of CORYLUS slowly increase during LPAZ III, reaching ~20 % at the zone boundary at 548 cm (~10,500 cal. BP). This increase reflects the early Holocene expansion of hazel in the study area. Across central and northern, the main expansion of hazel started roughly synchronous around 10,600 cal. BP (Giesecke et al. 2011). High-resolution pollen records from north-eastern Germany suggest some limited earlier expansion already at around 11,200 cal. BP (Theuerkauf et al. 2014), which is supported by the present findings. Moreover, both manual and automatic counts show early peaks in ULMUS and QUERCUS above 560 cm (~10,600 cal. BP). Whether these peaks reflect first small local populations of elm and oak, long distance transport events or still reworked material, cannot be solved with the data at hand. High resolution analysis from neighbouring sites may solve this question.

LPAZ IV – Early Holocene II (548 – 485 cm / 10,500 – 9500 cal. BP)
LPAZ IV covers the main increase in CORYLUS pollen percentages, i.e. the main expansion of hazel in the study region. The increase is overall steady, yet both manual and automatic counts show a short-lived delay at around 520 cm (~10,000 cal. BP), which coincides with somewhat higher values of PINUS, a first increase of QUERCUS, ULMUS and slightly later also of ALNUS above 1 % and somewhat lower values in WILD GRASS GROUP. Whether these changes reflect a regional scale vegetation response to, for example, a climatic event, or peculiarities of the study site, needs further evaluation. Among the non-pollen types, KM23 shows minor peaks in MICRO-CHARCOAL and in ROTIFERA EGGS. Changes in this NPP may reflect some changes in the lakes trophic state or water level. Above 515 cm (~9900 cal. BP), with further increasing CORYLUS values, ULMUS pollen percentages remain mostly well above 1 %, QUERCUS percentages at around 0.5 %.

Among the herbal pollen types, pollen percentages of WILD GRASS GROUP remain stable at about 2-4 % (mean = 2.7 %) throughout LPAZ IV, with, as mentioned above, slightly lower values only during the CORYLUS depression. ARTEMISIA (mean = 0.2 %) and FILIPENDULA (mean = 0.3 %) remain continuously present during the lower half of the zone, but are then rare or absent above

~530 cm (~10,250 cal. BP). Also CALLUNA VULGARIS is regularly present in this zone, yet rare (mean = 0.2 %). These observations suggest that patches of open vegetation likely persisted during that period.

NPPs are overall rare. In the middle of the zone, the values of ROTIFERA EGGS are somewhat elevated, with maxima around 2 %. Maxima of MICRO-CHARCOAL are at ~6 %.

LPAZ V – Mid Holocene I (485 – 385 cm / 9500 – 5900 cal. BP)

LPAZ V covers the main increase in the pollen percentages of warm-loving tree taxa – first ULMUS and QUERCUS, then ALNUS and with some delay TILIA and FRAXINUS. As mentioned before, ULMUS and QUERCUS are already continuously present with values around 1 % present since above 520 cm ~10,000 cal. BP), yet the main increase only starts at 485 cm (~9500 cal. BP), with ULMUS increasing somewhat faster than QUERCUS. Also ALNUS is continuously present with low values from ~10,000 cal. BP, the main increase starts at 465 cm (9200 cal. BP). For TILIA, first presence is indicated at 475 cm (~9300 cal. BP), the main increase starts at ~464 cm (~9200 cal. BP). FRAXINUS is continuously present above 447 cm (~8700 cal. BP), and the main increase starts at 432 cm (~8200 cal. BP). FRAXINUS pollen grains have been detected by TOFSI in several lower samples, for example more than 10 grains in each of the samples KM23B 454-455 cm (~8970 cal. BP), 464-465 cm (~9170 cal. BP), 480-481 cm (~9400 cal. BP) and 491-492 cm (~9600 cal. BP). These finds, which have been manually confirmed, may indicate that some small *Fraxinus excelsior* populations were present in the study region well before the main expansion at around 8200 cal. BP. Moreover, the main increase at around 8200 cal. BP coincides with a shift to higher pollen percentages in ALNUS and QUERCUS and to lower values in PINUS and CORYLUS, which points at some major changes at forest composition around that time.

Herbal pollen is overall rare, with some variation within the zone. Only WILD GRASS GROUP is continuously present, with a lower mean abundance (1.4 %) than in LPAZ IV (2.7 %). ARTEMISIA, CALLUNA VULGARIS, PLANTAGO LANCEOLATA TYPE and RUMEX have been detected discontinuously and with mostly very low percentages. PLANTAGO LANCEOLATA TYPE, commonly attributed to the plant species *Plantago lanceolata*, is one of the key land-use indicators. It is usually well present after the onset of the Neolithic land-use period(s), which in the study region starts after 6000 cal. BP (e.g. Feeser et al. 2019). The earlier occurrences of that pollen type in KM23, which have been confirmed manually, may derive from other *Plantago* species (Beug 2004). Pollen percentages of CALLUNA VULGARIS are highest from 430 to 410 cm (~8200-7200 cal. BP), with values of ~1 %. The manual counts of KM1 appear to show similarly high CALLUNA VULGARIS values only after 7000 cal. BP, which may be explained by the low sample resolution of KM1 in that section. Percentages of MONOLETE SPORES WITHOUT PERINE are also slightly higher in the period 8200-7200 cal. BP, and again towards the end of LPAZ V. They may indicate some expansion of wetland vegetation at the margins of the palaeolake.

Among the NPPs, KM23 shows elevated values of the green algae BOTRYOCOCCUS from 430 to 418 cm (~8050-7500 cal. BP) and PEDIASTRUM at 395 cm (~6500 cal. BP). MICRO-CHARCOAL values are markedly elevated from 415 to 395 cm (~8100 to 6500 cal. BP).

Overall, at 430 cm, i.e. at or shortly after 8200 cal. BP, KM23 shows changes in pollen and spore types from trees and herbs, as well as in NPPs. Moreover, lower Lycopodium percentages point at a temporarily increased pollen concentration. All these changes may relate to the 8.2 cooling event. However, as the age-depth model of the KM23 in this section is yet based on very view dating points, such interpretation must remain tentative.

LPAZ VI – Mid Holocene II (385 – 290 cm / 5900 – 3000 cal. BP)

The boundary between is LPAZ V and LPAZ VI is marked by the unmistakable ULMUS/elm decline. In the annually laminated record from Lake Belauer See, the decline occurred steadily from 6000-5800 cal. BP, with ULMUS values declining from ~10 % to only about 1 % (Dörfler et al. 2012). In KM23 the decline occurred instead very rapidly, i.e. ULMUS values declined from 9.4 % in sample 384-385 cm to 5.2 % in the consecutive sample 383-384 cm. In the following samples, ULMUS values decrease somewhat further to ~4 %. The decline is hence less pronounced in KM23. In the course of LPAZ VI, ULMUS values increase again to 5-6 % from 360 to 340 cm (~4800 to 4300 cal. BP) and finally decline to below 2 % in the top part of LPAZ VI.

Also other tree pollen types show variations within LPAZ VI. BETULA values show a minimum at 355 cm (~4800 cal. BP) and then slowly increase towards the upper zone boundary. CORYLUS values show a slightly earlier minimum at 360 cm (~4900 cal. BP), increase towards a peak at 325-315 cm (4000-3750 cal. BP) and then decline again. This peak occurs somewhat earlier in the manual counts of KM1 than in the automatic counts of KM23, which may point at dating uncertainties. QUERCUS and partly ULMUS behave conversely, i.e. they are higher at around 360-330 cm (~5000-4000 cal. BP) and at 305-295 cm (~3300-3000 cal. BP). Pollen percentages of FRAXINUS are initially low at ~3-4 %, then increase to a maximum of ~10 % at 360 cm (~4900 cal. BP) and then slowly decrease again to ~3-4 %. The peak is only present in KM23, not in the manual counts of KM1. A manual revision of the grains classified as FRAXINUS from selected samples with the highest FRAXINUS percentages confirms that these classifications are almost all correct. The peak may be absent from the manual counts due to the lower sample resolution of KM1. Finally, KM23 shows a first prominent peak of FAGUS, with values exceeding 2 % in the three consecutive samples from 380 to 377 cm. With the current age-depth model, these samples cover a period of 100 years, which about 100 years after the ULMUS decline. The peak is present in the two parallel cores KM23B and KM23C, and has been confirmed manually. After that peak, FAGUS is then again rare – values remain below 1 % until 297 cm (~3100 cal. BP). One interpretation of that peak is that beech did benefit from the widespread dying of elm, and established on the resulting open spots. However, apparently only one generation existed, i.e. beech could not regenerate in the then again closed forests. This hypothesis obviously requires that beech has been present in the region already before the elm decline. In KM23, FAGUS pollen is indeed recorded since ~8000 cal. BP with values of around 0.2 %. Such low values are often interpreted as long distance transport, yet they may also indicate rare regional presence.

In LPAZ VI, herbal pollen percentages are higher than before. WILD GRASS GROUP steadily increases from ~2 to 5 %. ARTEMISIA, PLANTAGO LANCEOLATA TYPE and RUMEX are present throughout LPAZ VI, yet discontinuously and with low values only. All three types show somewhat elevated values near the end of the zone, above ~300 cm (~3200 cal. BP). CALLUNA VULGARIS, which may derive from wetland rather than from upland vegetation, is continuously present in LPAZ VI, with values range from ~0.5 to 2 %. Also MONOLETE SPORES WITHOUT PERINE and SPHAGNUM spores are regularly present, yet with values below 1 %.

Among the NPPs, the two green algae BOTRYOCOCCUS and PEDIASTRUM are more abundant than before, particularly above 340 cm (~4400 cal. BP). Manual revision shows that the first prominent PEDIASTRUM peak at 356-357 cm (~4800 cal. BP) is incorrect. In this sample, amorphous objects were misclassified as PEDIASTRUM. Manual evaluation of every fifth sample shows that apart from this peak, other finds of BOTRYOCOCCUS and PEDIASTRUM are mostly correct. MICRO-CHARCOAL values increase above 368 cm (~5250 cal. BP) and then again above 318 cm (~3750 cal. BP). Trends in MICRO-CHARCOAL and, less clearly, in the green algae, are similar to the trends in NAP upland pollen. The values of ROTIFER EGGS are elevated around 362 cm (~5000 cal. BP) and around 310 cm (3500 cal. BP). Manual evaluation confirms that all these peaks are correct.

LPAZ VII – Late Holocene (290 – 194 cm / 3000 – 1000 cal. BP)
The most prominent feature of LPAZ VII is the stepwise increase in FAGUS and CARPINUS. Both pollen types start from very low values of only ~0.5 to 1 % at the lower zone boundary. They then show several peaks from 260 until 230 cm (~2300-1400 cal. BP). For FAGUS, four peaks approach ~6 %. With the exception of the second peak at 254-255 cm (~2150 cal. BP), all peaks clearly correlate with minima in WILD GRASS GROUP and other open indicators. CARPINUS shows similar, but slightly earlier and less pronounced peaks with maxima of ~1.5 %. Both pollen types then steadily increase from 230 cm until 220 cm (~1550-1300 cal. BP) towards maxima of ~15 % and ~3.5 %, respectively, and then decline again above 203 cm (~1100 cal. BP). Obviously, the late Holocene expansion of *Fagus sylvatica* and *Carpinus betulus* in the study region followed a complex pattern, which was influenced by land use activities. Another prominent feature of

All other tree pollen types show small variations until 200 cm 200 cm (~1050 cal. BP). Namely PINUS and CORYLUS, and less clearly BETULA, ULMUS and TILIA, decline slightly. Towards the end of LPAZ VII, starting at 200 cm, pollen percentages of ALNUS temporarily drop sharply, most likely reflecting the widespread alder decline that has been previously described e.g. from neighbouring northern Poland and other Baltic regions (e.g. Latałowa et al. 2019). As the likely cause, Latałowa et al. (2019) discuss the outbreak of a disease following a series of flood and/or drought events. The event has been dated to 1100 cal. BP in Lake Belauer See (Dörfler et al. 2012). In KM23, the decline in ALNUS is synchronous to a decline in FAGUS and a prominent increase in NAP UPLAND, including major land use indicators. Using data from only KM23, it is not possible to evaluate whether the decline in alder in the study region was caused by the increased land use activity, whether the two changes were related to the same (climatic) factor, or whether the synchronisation is coincidental. Moreover, in KM23 the decline in ALNUS is also synchronous to a sharp increase in SPARGANIUM (to some extend wrongly classified as FRAXINUS!) and THELYPTERIS, and a decline in PEDIASTRUM. These and further factors point at the terrestrialisation of the Kieshofer Moor palaeolake. Whether the terrestrialisation has been triggered by a climatic event, as has been proposed for the synchronous alder decline, needs further evaluating. Alternatively, the lake basin may have been drained artificially.

In LPAZ VII, herbal pollen percentages are higher than in the previous zones, indicating overall higher land use activity. In the longer trend, NAP UPLAND increase until ~235 cm (~1700 cal. BP), slightly decreases until ~220 cm (~1300 cal. BP) and then again increases in the topmost samples of LPAZ VII. In addition to these longer trends, KM23 shows several short-term fluctuations, e.g. peaks at 245 cm (~1900 cal. BP) and at 235 cm (~1700 cal. BP), which coincide with minima namely in FAGUS pollen percentages. WILD GRASS GROUP is the most abundant herbal pollen type. Pollen percentages start from ~3 % at the lowermost samples and increasing to a mean of 5.7 % above 270 cm (~2500 cal. BP). As mentioned before, namely from 260 cm to 230 cm (~2300-1400 cal. BP), fluctuations in WILD GRASS GROUP mirror changes in FAGUS and, less clearly, CARPINUS. Also ARTEMISIA, CHENOPODIACEAE AND AMARANTHACEAE, PLANTAGO LANCEOLATA TYPE and RUMEX are continuously present in LPAZ VII, yet mostly with values below 1 %, except for the topmost samples. At least ARTEMISIA and RUMEX show peaks synchronous to those in WILD GRASS GROUP from 260 cm to 230 cm, hence likely reflecting periods of intensified land use. Moreover, also SECALE and CEREALIA UNDIFF. as well as FILIPENDULA and SINAPIS were regularly detected. It should be noted that the detection of all these pollen types is still experimental because the small amount of training images.

For CALLUNA VULGARIS, the mean value in LPAZ VII is much higher (4.3 %) than in LPAZ VI (1.2 %). The higher values may reflect some expansion of *Calluna vulgaris* at the margins of the palaeolake. Previous unpublished studies indicate that the terrestrialisation of some marginal sectors started already around 2500 cal. BP. This interpretation is supported by the synchronous increase of other wetland types, namely CYPERACEAE, MONOLETE SPORES WITHOUT PERINE and SPHAGNUM.

On the other hand, the higher CALLUNA VULGARIS values also coincide with higher values in land use indicators, namely in WILD GRASS GROUP, and in MICRO-CHARCOAL particles. They may hence alternatively reflect the expansion of heathlands with *Calluna vulgaris* on terrestrial sites.

Towards the end of LPAZ VII, most upland and wetland herb pollen and spore types increase sharply, likely reflecting intensified land use activity in the study region and the terrestrialisation of the Kieshofer Moor palaeolake. The increase in SPARGANIUM is underestimated, because, as mentioned before, this pollen types has to some extend been wrongly classified as FRAXINUS.

Among the NPPs, LPAZ VII shows much higher values of PEDIASTRUM but lower values of BOTRYOCOCCUS than LPAZ VI. A manual check in 10 samples confirms that the objects identified as PEDIASTRUM colonies were indeed classified correctly by TOFSI. Also MICRO-CHARCOAL particles are more abundant than before, namely from 280 cm until 230 cm (~2600-1500 cal. BP) and again in the uppermost samples. Again, a manual check in 10 samples confirms that these classifications are mostly correct. Trends in MICRO-CHARCOAL values are similar to those in NAP UPLAND, WILD GRASS GROUP and CALLUNA VULGARIS. Moreover, also short lived peaks in MICRO-CHARCOAL, namely at 245 cm and 235 cm are synchronous to before mentioned peaks in e.g. WILD GRASS GROUP. The values of ROTIFERA EGGS are clearly elevated from 260 cm to 280 cm (~2800 to 2300 cal. BP), yet rare in the other parts of LPAZ VII.

Towards the top of LPAZ VII, the green algae BOTRYOCOCCUS and PEDIASTRUM are rare, reflecting the terrestrialisation of the palaeolake. Instead TILLETIA and ASSULINA, two characteristic NPPs of acidic *Sphagnum* peat, are present.

Upper peat layer
At the coring site, the lake sediments of the Kieshofer Moor palaeolake are covered by about 2 m of *Sphagnum* rich peat. Despite weak to intermediate decomposition, this section was excluded from the present analysis for several reasons. First, retrieving complete cores from this sections is difficult because of long *Eriophorum* roots. Moreover, test samples show a very low pollen concentration, which points at temporarily very high peat accumulation rates, interrupted by stagnation periods. Hence, the overall peat sections most likely does not represent a continuous record of the past ~1000 years.

Summary
Palynology has a long history in North-eastern Germany. The first pollen diagram published was one from palaeolake Kieshofer Moor (von Bülow 1928). Since than, more than 50 long pollen records that cover the Lateglacial and Holocene have been presented from the region. Only about ten of these records have been prepared in high samples resolution, usually meaning sample distance of 5-10 cm, and have been independently, radiocarbon dated. From these records, the general pattern of the post-glacial vegetation history of the region is well known (e.g. Theuerkauf et al. 2024).

The present, automatically counted pollen record KM23 well depicts these known trends. Beyond that, Due to its exceptionally high, 1-cm sample resolution and often very high pollen sums, KM23 beyond that points a number of hitherto unknown details. For example, during the early Holocene, pollen of warm-loving tree taxa is well present in low values long before the main expansion of the respective taxa. This long tail points at a more complex expansion history of warm-loving tree taxa, which may have been present in small, early populations long before their main expansion. Further high resolution studies will be needed to confirm this pattern and to detect the likely location of these early populations. We hypothesise that these early Holocene expansion patterns were mainly

caused by a progressive warming, yet a comparison with detailed climate data will be needed to test this hypothesis.
A particular, and previously much debated case is the expansion history of beech and hornbeam. KM23 for the first time shows a prominent yet temporary mid-Holocene expansion of beech, about 100 years after the elm decline. We hypothesise that beech did benefit from forest openings created by the elm decline. Similar observations from further sites are needed to verify this pattern. At the moment, we can only speculate why beech did not establish permanently on these sites. For example, unfavourable climate feature such as regular late frost may have limited the competitiveness of beech, so that the beech populations remained restricted. The present high resolution analysis also reveals sharp alternations between FAGUS and NAP UPLAND peaks, which underline the previously observed link between beech and land use activities in northern central Europe.

To interpret the features mentioned above in-depth, further analysis with similarly high resolution will be needed from further sites in the region. The TOFSI approach now allows to perform such analysis with reasonable efforts.

References

Beug, H.-J. (2004). *Leitfaden der Pollenbestimmung für Mitteleuropa und angrenzende Gebiete*. Verlag Dr. Friedrich Pfeil.

de Klerk, P. (2002). Changing vegetation patterns in the Endinger Bruch area (Vorpommern, NE Germany) during the Weichselian Lateglacial and Early Holocene. *Review of Palaeobotany and Palynology*, *119*(3–4), 275–309. https://doi.org/10.1016/S0034-6667(01)00103-8

de Klerk, P. (2008). Patterns in vegetation and sedimentation during the Weichselian Late-glacial in north-eastern Germany. *Journal of Biogeography*, *35*(7), 1308–1322. https://doi.org/10.1111/j.1365-2699.2007.01866.x

de Klerk, P., Helbig, H., & Janke, W. (2008). Vegetation and environment in and around the Reinberg basin (Vorpommern, NE Germany) during the Weichselian late Pleniglacial, Lateglacial, and Early. *Acta Palaeobotanica*, *48*(2), 301–324.

Feeser, I., Dörfler, W., Kneisel, J., Hinz, M., & Dreibrodt, S. (2019). Human impact and population dynamics in the Neolithic and Bronze Age: Multi-proxy evidence from north-western Central Europe. *The Holocene*, *29*(10), 1596–1606. https://doi.org/10.1177/0959683619857223

Giesecke, T., Bennett, K. D., Birks, H. J. B., Bjune, A. E., Bozilova, E., Feurdean, A., Finsinger, W., Froyd, C., Pokorný, P., Rösch, M., Seppä, H., Tonkov, S., Valsecchi, V., & Wolters, S. (2011). The pace of Holocene vegetation change – testing for synchronous developments. *Quaternary Science Reviews*, *30*(19–20), 2805–2814. https://doi.org/10.1016/j.quascirev.2011.06.014

Joosten, H., & de Klerk, P. (2002). What's in a name? Some thoughts on pollen classification, identification, and nomenclature in Quaternary palynology. *Review of Palaeobotany and Palynology*, *122*, 29–45.

Latałowa, M., Święta-Musznicka, J., Słowiński, M., Pędziszewska, A., Noryśkiewicz, A. M., Zimny, M., Obremska, M., Ott, F., Stivrins, N., Pasanen, L., Ilvonen, L., Holmström, L., & Seppä, H. (2019). Abrupt Alnus population decline at the end of the first millennium CE in Europe – The event ecology, possible causes and implications. *The Holocene*, *29*(8), 1335–1349. https://doi.org/10.1177/0959683619846978

Theuerkauf, M., Bos, J. A. A., Jahns, S., Janke, W., Kuparinen, A., Stebich, M., & Joosten, H. (2014). Corylus expansion and persistent openness in the early Holocene vegetation of northern central Europe. *Quaternary Science Reviews*, *90*, 183–198. http://dx.doi.org/10.1016/j.quascirev.2014.03.002

Theuerkauf, M., De Klerk, P., & Michaelis, D. (2024). Östliches Jungmoränengebiet. In I. Feeser, W. Dörfler, M. Rösch, S. Jahns, S. Wolters, & F. Bittmann (Eds.), *Vegetationsgeschichte der Landschaften in Deutschland* (pp. 531–544). Springer Berlin Heidelberg. https://doi.org/10.1007/978-3-662-68936-3_61

Theuerkauf, M., Nehring, E., Gillert, A., Bodien, P. M., Hein, M., & Urban, B. (2024). First automatic size measurements for the separation of dwarf birch and tree birch pollen in MIS 6 to MIS 1 records from Northern Germany. *Ecology and Evolution*, *14*(6), e11510. https://doi.org/10.1002/ece3.11510

von Bülow, K. (1928). Die deutschen Moorprovinzen. *Jahrbuch Der Preußischen Geologischen Landesanstalt*, *49*(1), 207–219.

Pollen diagram KM23 from palaeolake Kieshofer Moor (North-eastern Germany)

Automatic pollen recognition with TOFSI

Pollen sum includes selcted tree (green) and herbal (orange) pollen types

5x exaggeration line

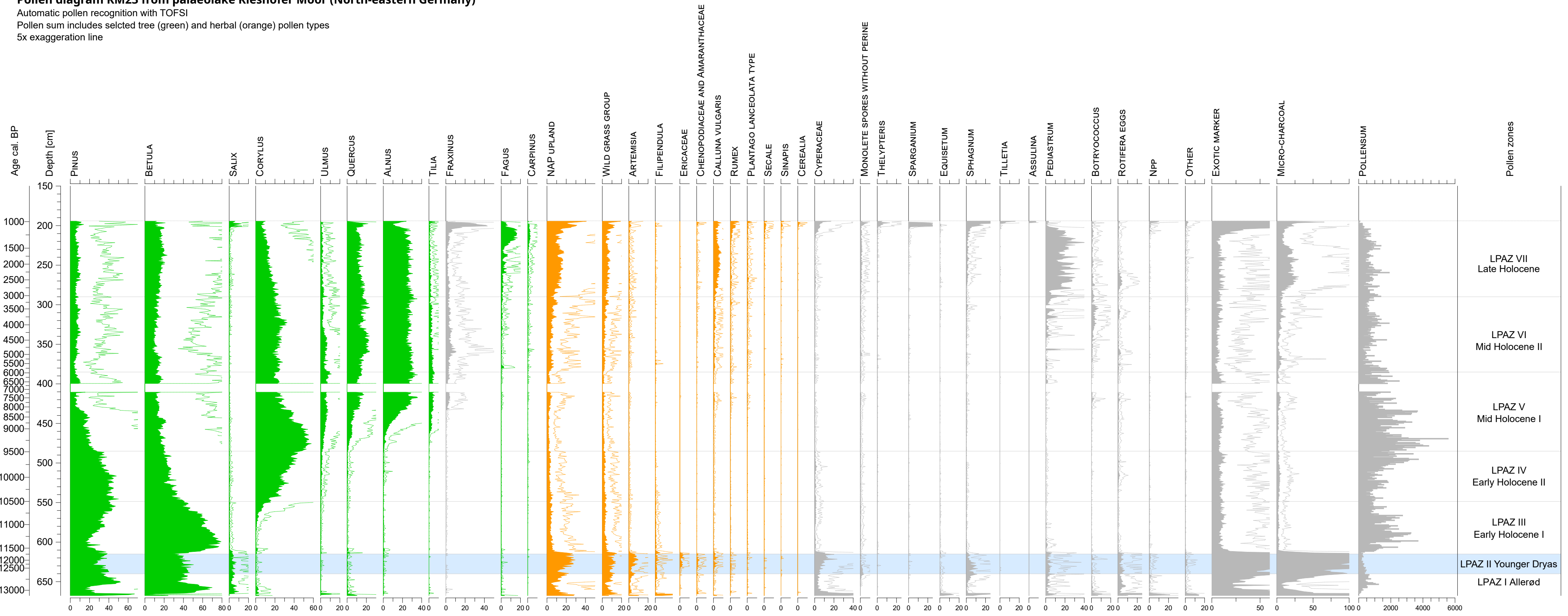

Pollen diagram KM1 from palaeolake Kieshofer Moor (North-eastern Germany)
Analysis: Martin Theuerkauf
Pollen sum includes selcted tree (green) and herbal (orange) pollen types
5x exaggeration line

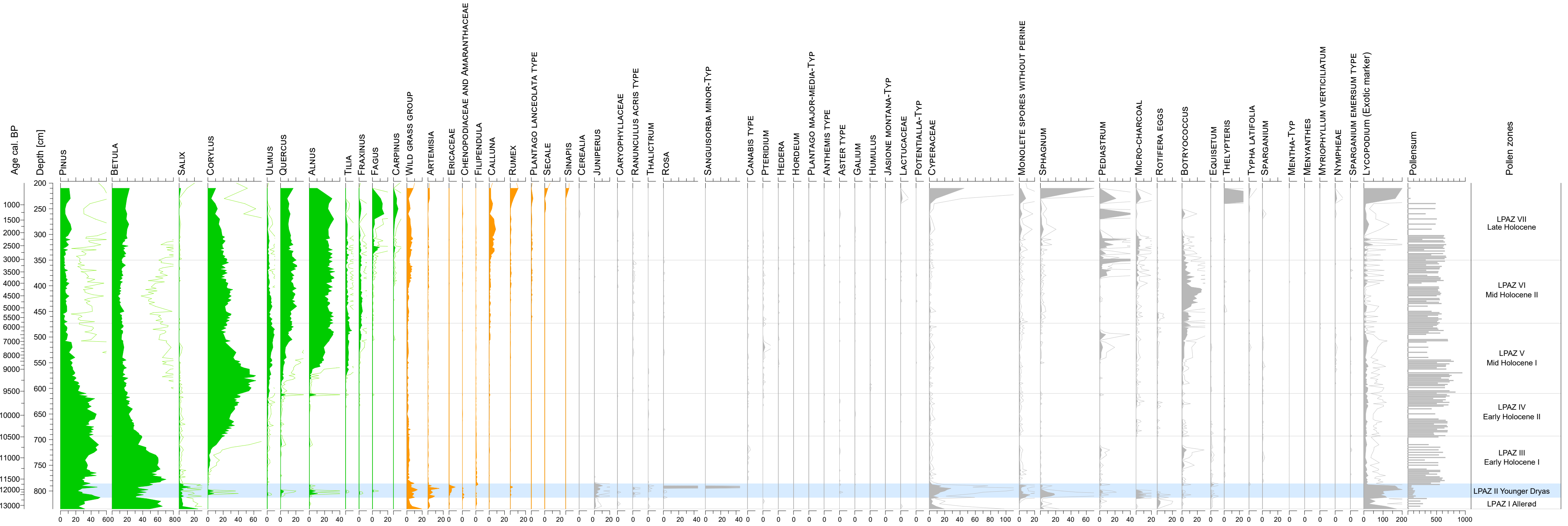

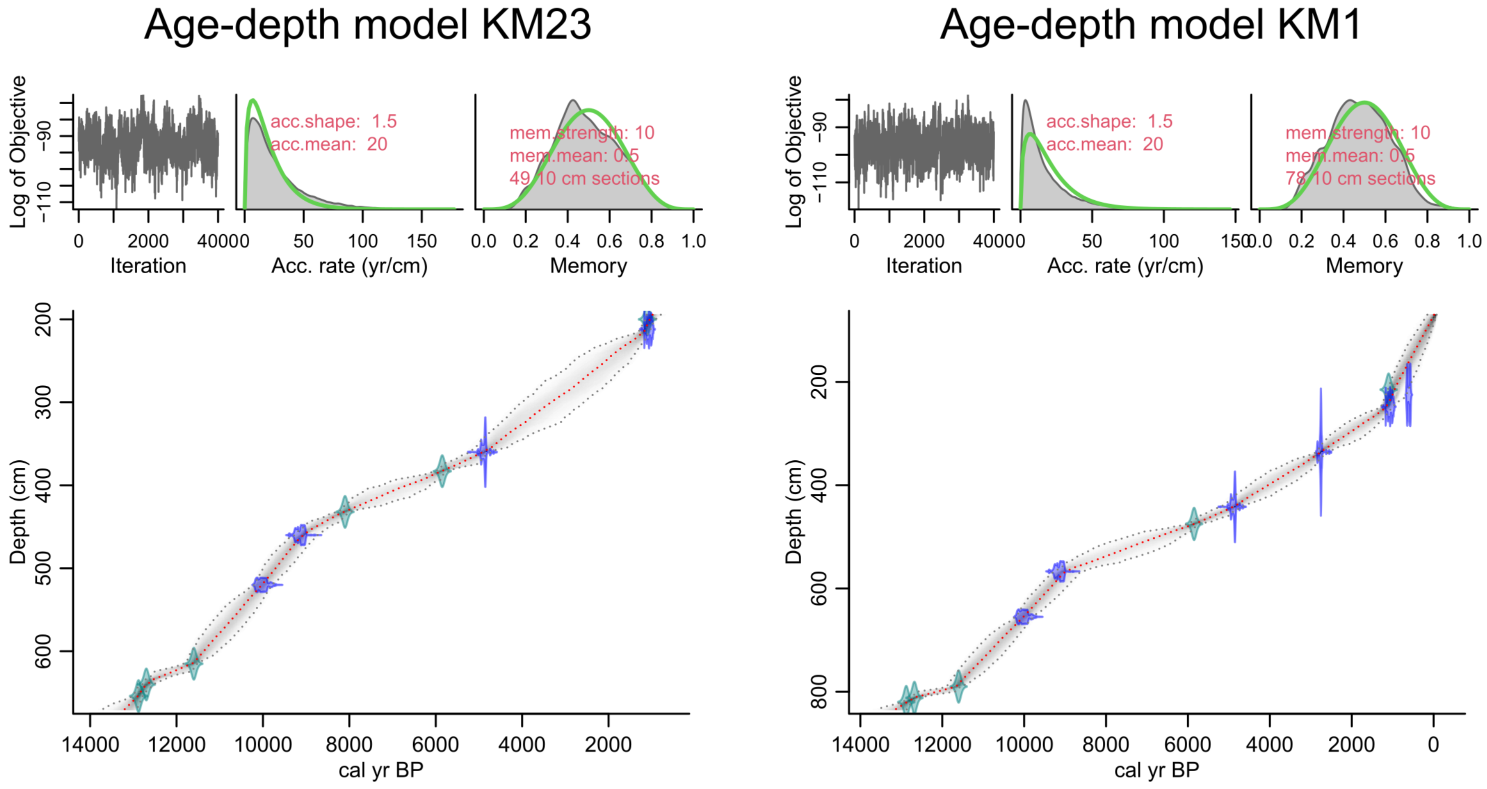
Age-depth model KM23
Age-depth model KM1
Log of Objective
Iteration
Acc. rate (yr/cm)
Memory
acc.shape: 1.5
acc.mean: 20
mem.strength: 10
mem.mean: 0.5
49 10 cm sections
78 10 cm sections
Depth (cm)
cal yr BP
